# Regulation of immunotherapeutic products for cancer and FDA’s role in product development and clinical evaluation

**DOI:** 10.1186/2051-1426-1-5

**Published:** 2013-05-29

**Authors:** Ramjay S Vatsan, Peter F Bross, Ke Liu, Marc Theoret, Angelo R De Claro, Jinhua Lu, Whitney Helms, Brian Niland, Syed R Husain, Raj K Puri

**Affiliations:** 1 Office of Cellular, Tissue, and Gene Therapies (OCTGT), Center for Biologics Evaluation and Research (CBER), Rockville, MD, USA; 2 Office of Hematology and Oncology Products (OHOP), Center for Drug Evaluation and Research (CDER), Food and Drug Administration (FDA), Rockville, MD, USA

## Abstract

Immunotherapeutics include drugs and biologics that render therapeutic benefit by harnessing the power of the immune system. The promise of immune-mediated therapies is target specificity with a consequent reduction in off-target side effects. Recent scientific advances have led to clinical trials of both active and passive immunotherapeutic products that have the potential to convert life-ending diseases into chronic but manageable conditions. Clinical trials investigating immunotherapeutics are ongoing with some trials at advanced stages of development. However, as with many products involving novel mechanisms of action, major regulatory and scientific issues arising with clinical use of immunotherapeutic products remain to be addressed. In this review, we address issues related to different immunotherapeutics and provide recommendations for the characterization and evaluation of these products during various stages of product and clinical development.

## Introduction

Since Coley’s observation in the 19^th^ century that some tumors respond to infectious challenges with streptococcus bacteria, medical science has been searching for a way to activate the immune system against cancer [1]. Coley's early studies eventually led to cancer immunotherapy using Bacillus Calmette-Guérin (BCG), which is still used to treat superficial bladder cancer [2].

Immunotherapeutic products can be classified broadly under (1) active immunotherapy (therapeutic vaccines), (2) adoptive cellular immunotherapy (transfer of immune cells [T and B cell therapies] or precursor cells [autologous or allogenic stem cell therapies] or the transfer of gene modified autologous or allogenic cells [Chimeric CAR/TCR engineered T cells]) or (3) passive immunotherapy (administration of antibody or receptor/ligand). These approaches are based on prior preclinical and clinical knowledge, as well as current understanding of immunology.

Of these three broad categories of immunotherapeutic products, adoptive cellular immunotherapy products are the most recent to show early signs of benefit and therapeutic value to the patient population [3]. The appeal of immune-mediated therapies is target specificity, with minimization of off-target side effects. However, immune-mediated therapies, which are intended to direct the immune system to counter tumors expressing certain tumor-associated antigens (TAA), may also induce a response to antigens that are expressed by normal tissues. Thus testing in appropriate preclinical studies is important to evaluate the safety of immune-mediated therapies. Some approaches to overcoming these challenges include developing a better understanding of the investigational immunotherapy products and their mechanism of action.

The Food and Drug Administration (FDA) regulates drugs and biologics under the authority granted to it by the Federal Food, Drug, and Cosmetic Act (FD&C Act, 1938) and its amendments [4].

Under this authority, FDA regulates the pre-market testing and marketing approval for all immunotherapeutic agents, either as drugs or biologics depending on the source and function of the investigational agent. Immunotherapeutic products that are regulated as biologics include antibodies and proteins and some nucleic acids. Other immunotherapeutic products that are biologics include live immunological cellular products (dendritic cells, T cells, etc., or their precursors [e.g., cord blood cells, adult stem cells], or cellular products pulsed with peptides), peptides or proteins acting as or mimicking tumor antigens to induce or boost a specific host immune response, and gene therapy products ^1^ (bacterial, viral and plasmid vectors, mRNAs, siRNAs, miRNAs, antisense RNAs, etc.). Immunotherapeutic products are regulated by the FDA under the purview of the Center for Biologics Evaluation and Research (CBER) Office of Cellular, Tissue and Gene Therapies (OCTGT) and by the Center for Drug Evaluation and Research (CDER), Office of Hematology and Oncology Products (OHOP) and Office of Biotechnology Products (OBP).

CDER is responsible for the review of monoclonal antibodies and proteins which are administered directly to patients for therapeutic intervention. These products include cytokines, (e.g., interferons), enzymes (e.g., thrombolytics), toxins, and all other proteins, except for those that are specifically assigned to CBER (e.g., vaccines and blood products). In addition, CDER is also responsible for the review of immunomodulators (non-vaccines and non-allergenic products intended to treat disease by inhibiting or modifying a pre-existing immune response).

CBER/OCTGT is responsible for the review of cancer vaccines and cell-based immunotherapeutic products. These products include dendritic cells, activated T lymphocytes (e.g., TIL, LAK), B cells, monocytes, cancer cells (chemically modified or unmodified), gene therapy products including e*x vivo* gene-modified cells, proteins and peptides as tumor antigens, either alone or mixed with adjuvants (e.g., KLH, BCG, GM-CSF), idiotypic and anti-idiotypic antibodies, and tumor cell lysates.

The development of immunotherapeutic products for cancer poses unique challenges and opportunities to the drug development process. In this review, we will outline FDA’s approaches to evaluating the safety and efficacy of these products using appropriate immunotherapeutic products as examples. This review will include discussions on requirements for product characterization, preclinical testing and clinical trial design, and clinical safety and efficacy testing for cancer therapeutic products, including cellular and gene therapy products. Various ways to comply with the Code of Federal Regulations (CFR) pertaining to biological therapeutic products (21 CFR 600–680 and 21 CFR 1270–1271) that require the investigational product to be well characterized (21 CFR 610), and free from extraneous material (21 CFR 610.13) and that the products be approved based on clinical determination of safety and efficacy (21 CFR 314.105) will also be discussed.

## Review

### Chemistry manufacturing and control (CMC) considerations

#### Product characterization

Product characterization is the evaluation of quality attributes of the product. A product should be sufficiently characterized so as to discern changes in product characteristics over time. Characterization of a product with regard to its critical quality attributes (CQA) and other quality attributes early in product development will assist in future comparability studies necessitated by process/manufacturing changes, thereby enabling faster product development. Evaluation of the CQA depends on a thorough understanding of the biology of the investigational product.

Adequate product characterization may pose extensive challenges for complex cellular biological products. An immediate challenge is the inherent biological variability of cellular products, and the extent to which this variability can be controlled on a lot-to-lot basis. The challenges are complicated by the fact that the early development of cellular products may be based on a yet incomplete understanding of the biological roles of all the product components and on the limited information gleaned from preclinical studies. Here we will discuss, with appropriate product examples, a way to achieve a more complete understanding of cell and gene therapy products including cancer vaccines and immunotherapeutics, through comprehensive analysis rather than a minimalistic approach to product characterization. The assessment of product quality attributes for well-characterized immunotherapeutic proteins reviewed by CDER will not be discussed in this review and readers are encouraged to review FDA guidance documents published on these topics [5-7].

### Early-phase product development

**Figure 1:**
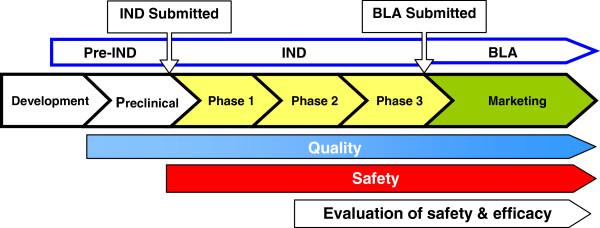
Biological product development overview.

Prior to initiating first-in-human, dose-finding (Phase 1) clinical studies under an Investigational New Drug (IND) application, preliminary specifications for product characterization should be in place. These product release specifications for immunotherapeutic products are established based on the IND sponsor’s previous experience with their product (and similar products, if applicable) and include analytical procedures based on CFR requirements. As product development proceeds, additional, and narrower, specifications for product quality and manufacturing consistency should be implemented based on the data obtained (Figure 1). At the time of initiation of clinical trials intended to support marketing applications (Phase 3), lot-release and other product specifications should be based on all information collected during product development, and consistent with data generated during clinical studies. During the conduct of Phase 3 trials, validation of analytical procedures for product testing should be ongoing or completed.

The regulatory requirements for biological product characterization include appropriate tests for (1) identity, (2) purity, (3) safety, and (4) potency. Over the years, FDA has published a number of guidance documents, to which the reader may refer for additional detailed information [5-9].

#### Identity

The identity of the final biologic product must be verified by assays that will identify the product for proper labeling and will distinguish the product from other products being manufactured in the same facility (21 CFR 610.14). Some examples of potentially useful targets for identity assays include cell surface markers, major histocompatability complex (MHC) antigen markers, gene expression, genetic polymorphisms, secreted molecules, and peptide sequences.

Identity assays should be specific and indicative of the nature or composition of the biological product. As an example, the use of T cell surface molecule CD4 will, by itself, be an insufficient T cell product marker given the current understanding in the field of immunology that CD4^+^ T cells encompass a wide array of effector populations. For example regulatory T cells (Tregs) are a subset of CD4^+^ T cells that also express FoxP3 and CD25, and could be detrimental components of a cancer immunotherapy product designed to elicit an anti-tumor response. Differences in the cellular sub-populations would not be obvious if the lot release was based on a flow cytometry assay that only enumerates the number of CD4^+^ cells. Additional information like the specificity of the CD4^+^ cells, and the population of memory or effector T cell sub-populations included in the product might also be important for final product characterization. Due consideration should also be given to minority cell populations in the final product as a purity measurement as discussed in the next section. If the acceptance criterion is based on the percentage of CD4^+^ T cells in a product, ascertainment of the phenotypic profiles of the remaining cells could provide additional important information on product characterization.

There are different considerations for the adequate identification of non-cellular immunotherapeutic products. For example, tumor antigen preparations encompass a wide variety of agents capable of interacting with the immune system to produce local inflammation, delayed type hypersensitivity reactions and ideally therapeutic effects such as tumor regression. Peptide and protein antigens designed to elicit direct immune responses against TAAs, have been used either alone, as conjugates with an immunogenic carrier protein (immunoconjugates) or as an integrated part of fusion proteins. Identification of these types of products includes evaluation of their primary, secondary and tertiary structures. Ideal peptide, protein, and immunoconjugate cancer vaccine therapeutics would include molecules where the size, structure and function can be well-controlled, and the upper and lower acceptance limits can be established and achieved in a consistent and reproducible manner.

#### Purity

Product purity (21 CFR 610.13) testing includes assays for pyrogenicity/endotoxin and for contaminants such as unintended cell populations (e.g., distinguished by phenotypes), residual proteins or peptides used to stimulate or pulse cells, and materials used during the manufacturing process, such as cytokines, growth factors, antibodies, and serum in cell therapy products or residual solvents in peptide vaccines. Acceptance criteria should be established for known impurities; for example, limits should be set for unintended cell types in the final cellular product.

Biological products intended for injection must be tested for pyrogenic substances (21 CFR 610.13(b)). Endotoxin testing using the Limulus Amebocyte Lysate (LAL) assay method has been successfully used in a number of early-phase clinical trials. Samples for purity testing should be taken from the final product, i.e., following final manipulations. FDA’s guidances recommend that an endotoxin acceptance criteria be set at or below 5 EU/kg body weight/hr for parenteral drug, except that intrathecally administered drugs have a limit of 0.2 EU/kg body weight/dose [10].

#### Viability

A minimum viability release criterion should be established for cellular immunotherapeutics. FDA’s guidances [11] recommend that this specification be at least 70% for products administered by the intravenous route of administration. If this level cannot be achieved, data should be submitted to support an appropriate level. Acceptance criteria for the final product should include specifications for the total number of viable cells and cellular sub-populations with documentation to support safety of the proposed cell number/dose.

#### Potency

Potency is defined as “the specific ability or capacity of the product, as indicated by appropriate laboratory tests or by adequately controlled clinical data obtained through the administration of the product in the manner intended, to effect a given result” (21 CFR 600.3(s)). FDA has published a guidance [12] for cellular and gene therapy (CGT) products to be administered under an IND or marketed under a Biologics License Application (BLA), which includes recommendations for developing tests to measure potency.

Potency assays, along with a number of other assays, are performed as part of product conformance testing (includes in-process, drug substance, and final product tests), comparability studies [13], and stability testing [14]. Potency measurements are used to ensure that only product lots that meet defined specifications or acceptance criteria are administered during all phases of clinical investigation and following market approval.

FDA recommends that manufacturers collect sufficient product characterization data (i.e., molecular, biochemical, immunologic, phenotypic, physical and biological properties) throughout preclinical and clinical development to provide support for the relevance and appropriateness of the chosen potency assay(s).

Ideally, a validated potency assay should be appropriately designed for each product based on a defined biological effect that closely reflects the proposed mechanism(s) of action/clinical pharmacodynamic response. FDA regulations allow for considerable flexibility in determining the appropriate measurement(s) of potency for each product. Because of the complex nature of CGT products, FDA recommends an incremental approach to the implementation of potency tests. FDA realizes the potency assay may change significantly during product development. It is scientifically challenging to identify appropriate potency assays for cell-based immunotherapeutic products since the active ingredient is often composed of whole cells and the activity of these products can generally not be attributed to one specific cell characteristic. It may be scientifically challenging to identify appropriate potency assays for cell-based immunotherapeutic products, for example, if the active ingredient is composed of a mixed population of cells and the activity of these products can't be attributed to one specific cell characteristic. FDA thus encourages IND sponsors to develop multiple assays at early stages of product development, so that as experience with a product accumulates, the sponsor has greater flexibility to select the assay(s) most indicative of potency. A timely discussion with the FDA review team is recommended as one designs, evaluates, and validates potency assays.

No single potency assay can adequately measure the CGT product attributes that will predict clinical efficacy. In general, the evidence of clinical efficacy is obtained from adequate and well-controlled investigations conducted with a consistently manufactured product, as specified in 21 CFR 314.126(d). Efficacy data from well-controlled clinical investigations suggests that a product has biological activity and thus is potent. Clinical data may be used to establish a correlation between biological activity and a more practical potency assay that can be used for lot release, stability, and/or comparability studies.

The potency of cell-based immunotherapeutic products can be measured using *in vitro* or *in vivo* tests, or both. For complex products consisting of more than one active ingredient (e.g., cellular tumor vaccines), FDA may require more than one assay to demonstrate the potency of each ingredient.

FDA recommends that a manufacturer collect sufficient product characterization data (i.e., molecular, biochemical, immunologic, phenotypic, physical and biological properties) throughout preclinical and clinical development that may provide support in developing an appropriate potency assay. Initial exploratory studies may help in assessing which product attribute(s) best correlate(s) with potency.

Many CGT products are multifaceted and have incompletely characterized mechanisms of action (MOA), making it difficult to determine essential product quality attributes, including potency. The MOA for CGT products may be dependent on more than one active ingredient (e.g., multiple cell types, multi-epitope vaccines).

The traditional approach for assessing the potency of biological products is to develop a quantitative biological assay (bioassay) that measures the activity of the product related to its specific ability to effect a given result. Bioassays can include *in vivo* animal studies, *in vitro* cell culture systems, or any combination of these. In cases where development of a suitable bioassay is not feasible, a surrogate measurement can be substantiated by showing a correlation with relevant product-specific biological activity. As development proceeds, clinical efficacy data may be used to establish a correlation between biological activity and a more practical potency assay that can be used for lot release, stability, and/or comparability studies. For many cases, a single biological or analytical assay may not provide an adequate measure of potency; FDA encourages developing complementary assays that measure different product attributes associated with quality, consistency and stability. These complementary assays might consist of a combination of biological assays, biological and analytical assays, or analytical assays alone [15].

FDA regulations require that product characterization for biologic products, as described in a BLA, include one or more validated potency assays. Numerous resources are available for analytical methods validation [16,17]. The validation process identifies potential sources of error and quantifies them within the assay method. The validation of the potency assay should include accuracy, precision (repeatability, intermediate precision), specificity, linearity and range, system suitability, and robustness. It is critically important to apply sound and appropriate statistical methods to the design and analysis of potency measurements. A timely discussion with the FDA review team is highly recommended as one designs, evaluates and validates potency assays.

### Late-phase product development

Late-phase product development typically involves the manufacture of the test article for evaluation in multicenter clinical trials that involve a few hundred to a few thousand study subjects. For this reason, product manufacturing is scaled up for the Phase 3, hypothesis-testing trials and for the potential marketing of the product, as changes are more easily addressed at this stage than after the conduct of Phase 3 clinical trials. Introduction of manufacturing changes following the conduct of Phase 3 clinical trials establishing clinical safety and efficacy may result in a need to bridge between the product used in clinical trials and the “to-be-marketed” product. Such bridging may include preclinical and clinical comparability studies that are necessary to support, thus delaying, the BLA. The most frequently encountered issues with production scale-up pertain to: 1) changes of manufacturing facilities, 2) changes of equipment related to growth, processing, and storage and 3) process changes. A change in manufacturing facilities requires a reevaluation of the facilities with reference to prevention of microbial contaminations, flow of materials, and personnel. Equipment changes are common as equipment used for small clinical trials may not be suitable for large scale manufacture. Changes in equipment may be closely related to a need for process changes; for example, while sucrose centrifugation-based viral vector processing may be sufficient for a small exploratory clinical trial, it may not be practical for the amount of product needed in a confirmatory trial. In another example, the use of different filters in the purification process may lead to a need to reevaluate the leachables and extractables profile. Process changes in general can result in changes in the impurity profile of the product (e.g., host cell proteins, residual host cell DNA), requiring further changes in the manufacturing process. Additionally, changes in production timing may lead to the introduction of intermediate storage steps in the manufacturing cycle, which may require additional tests for stability and sterility. While these examples of manufacturing changes may not be exhaustive, all manufacturing changes will require evaluation of both their immediate (e.g., changes in impurities profile) and downstream effects (e.g., changes in immunogenicity of the product). For these reasons, FDA recommends that the IND sponsors contact FDA’s product review offices (OCTGT or OBP, as appropriate) prior to implementing substantive manufacturing changes and prior to the initiation of Phase 3 trials intended to establish safety and efficacy for a marketing application.

Although complete identity and potency tests are not required during the early stages of immunotherapeutic product development, they are required prior to initiation of Phase 3 studies. To support late-phase biological product development, identity, purity, and potency assays should be established during the early clinical trials (Phase 1 and 2). The removal of all in-process reagents, ancillary factors such as cytokines, growth factors, antibodies, or enzymes to acceptable levels should either be validated or specifications set for maximum acceptable limits before the initiation of Phase 3 trials. Stability data to support the proposed dating period for Phase 3 clinical studies should also be available at this time. The stability program should address identity, purity, and potency of the product and intermediates during storage, and should be initiated as soon as possible in order to provide real time stability data for products where an extended dating period is desired. The integrity of the product could include measurement of viability and phenotype of cellular products. FDA also recommends that an end-of-phase 2 meeting be scheduled with the Agency to identify and discuss all outstanding chemistry, manufacturing and control issues for the product.

#### Control of product variability

Product variability is inherent in biological products and the variability has to be controlled, with the knowledge that as the manufacturing process evolves through various phases of the clinical evaluation and manufacturing scale-up, the product may manifest changes.

#### Quality of raw materials and reagents

In addition to changes in the product resulting from changes to the manufacturing process or growth conditions discussed above, product changes may also be introduced by the starting materials, and reagents. To control change, vendor qualifications should be built into the quality assurance and quality control (QA/QC) program early in the product development cycle [11]. The safety of raw materials and reagents should be ensured prior to introduction into the manufacturing stream. Strategies for the control of product heterogeneity have to be developed and product should be tested regularly during development. Assessment of change should include a comparability study conducted using well characterized reference materials.

Procedures used in manufacture should be refined during early-phase studies, but the manufacturing process should be well established prior to initiating Phase 3 studies, and changes to the process should be minimized once the Phase 3 trials are initiated. Changes in manufacturing during or after the Phase 3 studies will require demonstration of product comparability, which may include preclinical and or bridging clinical studies.

#### Reference materials

Reference materials (RMs) are used for assay development and assay validations. One type of RM is a product sample that is well characterized with reference to its essential quality attributes. Frequently these reference samples are products from early production lots, that have been extensively evaluated by appropriate analytical methods (e.g., analysis for cell surface markers, cell number, viability), and biological methods (e.g., biological function assays such as immunogenicity, tetramer binding, viral titer, etc.). When qualified assays are used to evaluate a product from an early production lot, that product may in turn be used as an in-house reference material for purposes such as evaluating product comparability after manufacturing changes or product scale-up during later phases of the product development cycle. In such cases it is essential that there be sufficient product manufactured for the specific purpose of use as a reference material. Biological RM rederived from a RM may pose a risk of “product drift” with each successive lot, that while in themselves may remain within their product specifications, could constitute a large and unacceptable variant from the original product. To ensure that a RM continues to be valid, a RM re-verification process to demonstrate that a RM is still fit for this purpose should be established [18] .

FDA continues to actively participate in reference method development activities and liaise with standard setting organizations such as American Society of Testing and Materials International Organization of Standardization, American Type Cell Collection, and other organizations (e.g., World Health Organization, International Conference on Harmonization, National Institute for Biological Standards and Control) to promote good manufacturing practices and provide methods and reference materials for testing and analyses that support characterization of biological products [19,20]. Biological reference materials are complex and are affected by various parameters, such as growth and storage conditions, and thus require characterization that is based on their intended mode of action. A limited number of cell substrate and viral reference materials are currently available from ATCC [21,22].

#### Adjuvants

Vaccine adjuvants are constituent materials (21 CFR 610.15) that are evaluated in combination with an antigen and used to potentiate or augment immune responses to the target antigen. There are two adjuvants that are in use in FDA-approved vaccines: aluminum salts including aluminum hydroxide, aluminum phosphate, and alum (potassium aluminum sulfate), and ASO4, a mixture of aluminum salts and monophosphoryl lipid A (MPL) used in the FDA-approved cervical cancer vaccine Cervarix. Examples of aluminum salt adjuvanted vaccines include DTaP vaccines, pneumococcal conjugate vaccine, and hepatitis B vaccines. Vaccines containing aluminum salt adjuvant have a demonstrated safety profile with over six decades of use and have only uncommonly been associated with severe local reactions. In view of the number of aluminum salt containing vaccines that have become a part of standard childhood vaccination regimens, FDA has specified a limit to the amount of aluminum in vaccines of 0.85 mg/dose with some exceptions (21 CFR 610.15).

A number of prophylactic and therapeutic cancer vaccines undergoing evaluation in clinical trials are administered in conjunction with newer adjuvants such as MF59 (Oil-in-water emulsion), [23] saponin-based adjuvant QS21 [24], and adjuvants with microbial derivatives: AS15, containing MPL, QS21, CpG and liposome [25] . There is also interest in the use of human cytokines and growth factors, which are licensed for the treatment of various disease conditions, as potential adjuvants. Examples of approved cytokines and growth factors include IL-2 (aldesleukin [26]), GM-CSF [(granulocyte-macrophage colony stimulating factor (sargramostim)], and erythropoietin alfa. In addition, other cytokines and cytokine blockers aimed at redirecting immune responses are also currently being evaluated as adjuvants for immunotherapy applications [27] .

As constituent materials of the final product, adjuvants must meet generally accepted standards of purity and quality. A change in adjuvant during clinical development may constitute a change in product requiring a new IND. The FDA recommends contacting appropriate product offices at the FDA for additional clarity when considering such changes.

### General considerations and tests required during all stages of product development

Licensed biological products must meet specifications for appearance, identity, purity, safety, and potency. These product attributes are characterized and refined throughout product development. FDA approves BLAs based on substantial evidence of effectiveness and reasonable assurance of safety for the product’s intended use. While efficacy of the product is primarily established in clinical studies, an assurance of product safety is required during all stages of both clinical and product development. The following section explores product safety testing requirements for the clinical evaluation of investigational products.

#### Safety testing and acceptance criteria

Required product testing to assure the safety of biological products includes tests for (1) sterility, (2) mycoplasma, (3) adventitious viral agents, and (4) general safety. These tests are discussed in detail below and are described in Title 21 CFR Parts 211 and 610 and in FDA guidance documents. Final product release specifications for safety are required for all phases of IND submission. However, actual tests (such as the use of rapid microbial detection tests for sterility) and specifications may evolve during product development. Specifications used for product intermediates should also be reported, as appropriate to the IND. In addition to the final product testing, in-process testing can provide meaningful insights into safety of the final product, particularly for cellular products that have a short shelf life. All specifications should be clearly described in the IND submissions, and a tabular format should be used as appropriate.

#### Sterility

Sterility (bacterial and fungal) testing on the final product should be performed according to the requirements in 21 CFR 610.12. A 14-day direct inoculation test method as described in the United States Pharmacopoeia (USP) <71> is typically used to evaluate sterility of cellular immunotherapeutics. Alternatively, an automated detection or other method may be used if validated appropriately. As antibiotics present in cell culture test samples may confound sterility results, bacteriostasis and fungistasis testing (as described in USP <71>) should be performed to ensure that any residual antibiotic does not interfere with the sterility testing. Samples for sterility testing should be obtained after final product manipulation, i.e., after all washing procedures, and, preferably, as the final formulation. If the product is frozen prior to use, samples for sterility testing should be taken prior to cryopreservation. If the product is manipulated by washing, culture, etc. after thawing, or at any other time (e.g., after transport to the study site), the IND sponsor should repeat the sterility testing. In general, a “no growth” acceptance criterion is used for release of cellular products.

In contrast to other immunotherapeutic products, the results of a full 14-day sterility assay on the final cellular product may not be available prior to administration of the product; therefore, a sample including both cells and supernatant should be taken for sterility testing approximately 48–72 hours prior to the final harvest (or coincident with the last re-feeding of the culture). An interim reading of this sterility test at the time of product release will contribute to a sterility determination. A rapid microbial detection test, such as a Gram stain, should also be performed on the final product prior to administration, and these results will also contribute to a sterility determination. A sterility test should also be performed on a sample of the final product, and both the in-process (48–72 hour) and the final product sterility test should be continued for the full 14–day culture. Additionally, a positive sterility test action plan should be submitted that documents procedures to follow in the event that either of the 14-day sterility tests reveals that a contaminated product was administered to a patient. These procedures should include: physician and patient notification, identification and sensitivity testing of the contaminant, additional patient monitoring, investigation to determine potential sources of the contamination and corrective actions, and reporting of the incident to the IRB and FDA as an adverse event within 15 calendar days.

#### Mycoplasma

If product manufacture includes cell culture of more than 24 hours, the product should be tested for mycoplasma contamination. Mycoplasma testing should be performed on samples (including both cells and supernatant) obtained prior to final manipulation (i.e., on cells still in conditioned culture medium, before final harvest and wash). For the recommended testing procedure description, refer to the appropriate FDA guidance document [9]. For products that must be administered before obtaining mycoplasma culture test results, alternative rapid mycoplasma detection assays (e.g., PCR based assays) may be performed. For Licensure, equal sensitivity and specificity between the rapid assay and culture-based assay must be validated.

#### Adventitious agents testing

Adventitious agents (AA) are microorganisms that have been unintentionally introduced into the manufacturing process of a biological product. All biologically derived starting materials, including cell substrates, viral banks, growth media and components such as fetal bovine serum (FBS) need to be screened and or tested for the presence of AA. All cell and viral banks are now routinely tested for known viral AA (see Table 1). The challenge is to test for the presence of human or zoonotic viral agents that have not yet been identified or agents that cannot be assayed by a reliable test (such as vCJD). In order to ensure that a product is free from AA, existing guidances emphasize the need to use multiple strategies and tests such as *in vitro* assays, *in vivo* assays, reverse transcriptase assays, transmission electron microscopy (TEM), as well as sourcing materials that are known to be free of specific pathogens (e.g., specific Pathogen Free flock-derived chicken embryonic fibroblastic cells; Fetal Bovine Serum sourced from BSE free countries). A number of specific assays have been developed to detect many known viruses in cell substrates, end of production cells and final product (e.g., ELISA and NAT based assays such as PCR, qPCR, antibody production tests). A number of non-specific assays are also employed in assaying for the presence of unknown viruses in cell substrates such as cell co-culture tests, and genome and transcriptome analysis approaches. Among the non-specific assays that have been successfully employed to detect viruses in final products is the whole genome high throughput sequencing approach that led to the detection of Porcine Circovirus DNA-1 in vaccine preparations [28].

**Table 1:** A list of viral agents that should be tested in human cell lines used to manufacture cancer immunotherapeutic products

Human Viruses:	Cytomegalovirus (CMV)
	HIV-1 & 2
	HTLV-1 & 2
	Epstein-Barr Virus (EBV)
	Hepatitis B Virus
	Hepatitis C Virus
	Human Parvovirus B19
	Adenovirus
	Adeno-Associated Virus (AAV)
	Production cell medium/reagents Specific Viruses (e.g., Bovine and Porcine Viruses)

A general safety test performed by injecting guinea pigs with the investigational agent is also required (21 CFR 610.11) as a lot-release test for marketed biologic products. If it is impractical to include this general safety test in release testing, the FDA can grant exemptions to this test requirement as provided for under 21 CFR 610.11(g).

### Preclinical evaluation

#### General considerations for the preclinical assessment of immunotherapy products

Considering the range and complexity of immunotherapeutic products, no single preclinical program encompasses adequate evaluation of potential toxicities associated with the administration of every immunotherapeutic product in humans. Therefore, FDA has adopted a scientific data-driven, case-by-case approach to assess the safety of these products. This section will discuss some general principles for the preclinical evaluation of immunotherapeutic products, followed by a more detailed discussion of selected immunotherapeutic product types.

The overall goal of the preclinical studies for immunotherapeutic products is the same as that for other types of products: to provide data to support the safety of an investigational product in humans by defining its toxicological and pharmacologic characteristics. Safety concerns for these products can exist at multiple levels, including those that are related to the product (e.g., the replication of a viral vector *in vivo* or autoimmunity due to a high homology between an immunogenic epitope and an endogenous target), the process (e.g., the introduction of adventitious agents, or cell transformation due to *ex vivo* manipulation), and the biological function (e.g., polarization of the immune system, or overstimulation of the immune system due to immunomodulation). Data from *in vitro* and *in vivo* preclinical studies, conducted in conjunction with appropriate product characterization testing, help determine an acceptable safety profile for an immunotherapeutic product.

Federal regulations require the submission of “adequate information about the pharmacological and toxicological studies … on the basis of which the sponsor has concluded that it is reasonably safe to conduct the proposed clinical investigations.” The regulations further stipulate that “the kind, duration and scope of animal and other tests required varies with the duration and nature of the proposed clinical investigations” [21 CFR 312.23 (a) (8)]. Practically, preclinical studies conducted to support immunotherapeutic product development serve to: 1) identify potential target organs/tissues of toxicity and determine if these toxicities are reversible; 2) identify an appropriate starting dose level and inform the dose-escalation scheme and the dosing regimen of a first-in-human trial; and 3) identify parameters for safety and activity monitoring in humans.

To achieve these objectives, the conduct of *in vitro* and *in vivo* studies designed to define and understand the pharmacological properties of the immunotherapeutic product are an important first step. These proof-of-concept studies, together with prior knowledge of the targeted immune pathway and of related or similar products, help address several objectives: 1) establish a scientific basis for conducting a specific clinical trial, 2) determine a minimal pharmacologically effective dose level and immunization regimen, 3) characterize a potential dose–response relationship, 4) optimize the route of product administration, and 5) provide the basis for the animal species or animal disease model(s) used for further preclinical testing. Characterizing the humoral and cellular immune response in animals exposed to the immunotherapeutic product, with or without various adjuvants, helps to address many of these objectives. The immune system, in general, is designed to respond quickly to internal and external stimuli. Thus products targeting the immune system can have rapid and unintended effects. For example, a change in the concentration of an immunomodulatory agent can have several different effects on cells. Therefore, it is important for the animal studies to include adequate evaluation of the immune response to the administered product. In addition, correlation of an immune response with functional outcome, such as an anti-tumor response, improvement of disease status, or improved survival, in a tumor-bearing animal model is desired.

The primary objective of the toxicology studies is to identify potential adverse findings resulting from administration of biologically active dose levels of the immunotherapeutic product. Many preclinical studies are conducted to support a first-in-human clinical trial. Thus, understanding the relationship between dose level and toxicity in these early studies, if any, is important, as these data will help establish a safe starting dose level, dosing route, dosing schedule, and dose-escalation scheme for a clinical trial. This information can also help define subject eligibility criteria and determine appropriate clinical monitoring following product administration. The toxicology studies will include traditional endpoints, such as mortality, clinical observations, body weights, clinical pathology, and histopathology. Each endpoint provides insight into the safety profile of the immunotherapeutic product. For example, clinical pathology, which may include serum chemistry, hematology, coagulation, and urinalysis parameters, is a nonterminal assessment of the functional status of major organ systems. Histopathology evaluation, including local tissue reactivity, is a terminal analysis that can further evaluate both organ function and architecture, as well as potentially provide some understanding as to the mechanism of any toxicities observed. Depending on the type of immunotherapeutic product under investigation, additional endpoints, such as toxicokinetics or immunotoxicity, may be included in general toxicology studies or assessed in independent studies to address potential safety concerns. Additional studies, such as developmental/reproductive toxicology studies, may be needed as the immunotherapeutic product progresses to late-stage clinical trials, or if there are significant modifications to the immunotherapeutic product.

Basic study design considerations can also greatly enhance the quality and interpretation of the resulting preclinical data. To the extent possible, a nonbiased study design incorporating elements such as randomization of study animals according to a prespecified method, and assessment of certain study parameters, such as histopathology, is important. Other important design features include: 1) adequate numbers of animals for statistical analysis, 2) appropriate control groups, 3) multiple dose levels of the immunotherapeutic product, 4) a dosing regimen and route of administration similar to those planned for the clinical trials, and 5) adequate study duration to allow for comprehensive assessment of potential adverse findings. The pivotal toxicology studies should be conducted in compliance with Good Laboratory Practice (GLP) regulations (21 CFR Part 58), to assure study integrity.

The interpretability of the resulting pharmacology and toxicology data depends on the biological relevance of the animal species used. The immunotherapeutic product should be pharmacologically active in the species, and the target antigen expression pattern should be similar between animal and human. When administration of the investigational product in various animal species will not yield informative data, use of an animal analogue may be appropriate. Toxicology studies are generally conducted in healthy animals that usually offer the advantages of extensive historical control data and availability of sufficient numbers of animals. A ‘hybrid’ study design, using an animal model of disease, that incorporates both activity and safety endpoints in a single study can, however, sometimes be a more informative approach, and has been applied to preclinical evaluation of some immunotherapeutic products. Consultation with the appropriate center (CBER or CDER) and division within the FDA to discuss the appropriate animal species and models to use in a preclinical testing program for a specific immunotherapeutic product is encouraged.

### Preclinical program designs for specific immunotherapeutic products intended to treat cancer

#### Small molecule and biotechnology-derived pharmaceuticals

In general, requirements for the preclinical evaluation of small molecule or biotechnology-derived products specifically targeting the immune system and/or its regulation are the same as for other small molecule or biotechnology-derived products with non-immune targets. As a starting point, toxicology studies should be conducted in two species, one rodent and one non-rodent. If these standard studies are unlikely to yield relevant data, other approaches might be justifiable. For example, for many biologic products, such as monoclonal antibodies, there may be no relevant rodent species; thus, a single non-rodent species for toxicological evaluation may be justified. For the development of pharmaceuticals targeting the immune system for the treatment of cancer, the guidelines outlined in the International Conference on Harmonisation (ICH) Guidances for Industry S9: Nonclinical Evaluation of Anticancer Pharmaceuticals and S6: Preclinical Safety Evaluation of Biotechnology-Derived Pharmaceuticals, the S6 Addendum should be followed [29-31] . In addition, some consideration may be given to the ICH S8 guidance on immunotoxicity [32] . While this guidance is specifically concerned with unintended immunosuppression or immunoenhancement mediated by drugs, some of its recommendations may also be relevant to pharmaceuticals designed specifically to modulate immune function and may give developers an idea of the kinds of evaluations which would be useful to regulators. Many of the evaluations described in this guidance can be incorporated into standard toxicity studies, including detailed immunophenotyping and assessments of cytokine release following product administration. In addition, a potential strength of nonclinical evaluation of products with immune targets is the relative ease of obtaining primary human cells for *in vitro* testing. Immune assays using relevant primary human cells can serve as valuable tools for the prediction of potential toxicity, with results from these assays being used as indicators of both the primary clinical effects of the product and the relevance of the animal species used in toxicology studies. In addition, effects of a product on the function of primary human cells can be used in conjunction with traditional animal studies for the estimation of a minimally anticipated biological effect level (MABEL). The idea of the MABEL has been accepted under ICH S9 as a potentially useful measure for establishing an acceptable starting dose level in clinical oncology trials investigating products with immune agonistic activity, especially in cases where there is little experience with the targeted pathway or no relevant animal model. Other approaches to set a starting dose level may be acceptable as well.

#### Cell-based immunotherapeutic products

Many immunotherapeutic products are cell-based, and include autologous or allogeneic dendritic cells, natural killer (NK) cells, T cells, and tumor cells. A safety concern for some of the cell-based immunotherapeutic products is uncontrolled proliferation *in vivo*. A recent example is the persistence *in situ* of K562-GM-CSF cells (GM-CSF-expressing human leukemia cell line) after subcutaneous administration in humans even though the tumor cells were irradiated prior to inoculation. The prolonged expression of GM-CSF from these cells may have contributed to the leukocytosis observed in the patient [33]. In another case, following infusion in patients, the T cells expanded approximately 1000-fold or more, with concomitant significant increase in proinflammatory cytokine levels, and associated adverse effects [34]. Thus, preclinical studies for these products should assess the proliferation status and the potential for clonality of the cells through *in vitro* and/or *in vivo* testing. In addition, administration of a cytokine with a cell-based immunotherapeutic product may affect the *in vivo* function and safety of the cell-based product. The potential for such interactions should be considered when designing preclinical studies.

#### Gene-based immunotherapeutic products

Gene-based immunotherapeutic products include tumor antigens co-expressed with various transgenes such as cytokines and costimulatory factors, delivered by plasmid DNA, viral vectors, or microorganisms. This product class also includes genetically modified cells. The safety and potential efficacy of these products is influenced by the vector and by the expressed transgene(s). For example, in a clinical study evaluating the use of *ex vivo* engineered T cells for renal cell carcinoma, immune responses were detected to both the chimeric antigen receptor (CAR) and to the retroviral vector. These immune responses may have influenced the observed limited *in vivo* persistence of these CAR T cells [35]. Use of vectors that are constructed of a retroviral or lentiviral backbone may result in insertional mutagenesis, which raises tumorigenic concerns for some genetically modified cells, especially when these cells are expected to persist *in vivo* for a long time. In addition to the preclinical studies conducted to determine the *in vivo* safety of the administered vector and of the expressed transgene(s), characterization of the biodistribution profile of the vector to confirm its presence at the desired therapeutic sites(s), as well as its absence in non-target tissues, is important [36].

#### Therapeutic vaccines

The therapeutic vaccines discussed in this section consist of the conventional tumor associated antigen (TAA)-derived vaccines, such as synthetic peptides, purified recombinant antigen-based proteins, anti-tumor idiotypic antibodies, conjugated antigens, and tumor lysates. These antigens are used to induce an immune response against the tumor. Potential safety concerns include local inflammatory reactions, systemic toxicity, adverse effects on the host immune system, and autoimmune responses, which can be caused by impurities, contaminants, or the components of the vaccine formulation. Preclinical studies should be designed to address these concerns [37]. The selected antigen may be present only in the tumor cells (e.g., tumor-causing viral proteins or mutant proteins in tumor cells), which is desired, or it may be differentially expressed in tumor cells versus normal cells (e.g., cancer/testis antigens, CEA, and Her2/neu). An immune response to a tumor antigen is expected after immunization; however, a similar immune response to the same antigen in normal cells poses a potential safety issue. In addition to evaluation of this safety concern in animals when feasible, data generated from a tissue expression profile analysis can provide valuable information as to the possible normal tissues/organs that the selected antigen may also target. Thus, such an analysis can help determine subject eligibility criteria, as well as aspects of the clinical monitoring plan. Finally, the preclinical program for a therapeutic vaccine that may be administered with one or several adjuvants should evaluate the activity and safety of the vaccine-adjuvant combination.

#### Use of adjuvants in combination with immunotherapeutic products

An adjuvant is an agent that augments or directs the specific immune response to an antigen (in this case, the immunotherapeutic product). It is often co-mixed or co-administered with the antigen to induce a more robust immune response. Adjuvants act through diverse mechanisms, such as creating an antigen repository effect, interacting with specific innate immune pathways, and serving as ligands for different pattern recognition receptors. There are many different forms of adjuvants, including traditional aluminum salts (e.g., aluminum hydroxide, aluminum phosphate), lipopolysaccharides and their derivatives, cytokines, CpG oligodeoxynucleotide, and others. The adjuvant used determines, to some degree, the general type of immune response that will be generated by the immunotherapeutic product. Therefore, data to support the rationale for the adjuvant selected is important. Potential toxicities may be caused by the administration of the adjuvant alone (e.g., induction of hypersensitivity and autoimmunity; induction of proinflammatory effects), or by possible additive or synergistic effects when given in combination with the immunotherapeutic product. Therefore, preclinical testing should include the assessment of the safety and activity of the immunotherapeutic product alone, the adjuvant alone, and the immunotherapeutic product in combination with the adjuvant, administered according to the planned clinical immunization regimen and route of administration. For general principles regarding preclinical considerations for adjuvants in therapeutic vaccines, refer to the European Medicines Agency document ‘Guideline on Adjuvants in Vaccines for Human Use; January 2005 [38] .

### Clinical development and statistical assessment plans

Immunotherapeutic products, specifically monoclonal antibodies, have become part of the standard armamentarium of the practicing oncologist, with numerous indications and products licensed by FDA and international regulatory agencies. Therapeutic cancer vaccines utilizing active immunity have proved more challenging to develop, and to date only one product and therapeutic indication has been licensed by FDA. It is hoped that an understanding of the events generating and regulating tumor immunity will result in more licensed active cancer immunotherapies [39]. This section will address clinical trial designs, clinical safety, and clinical efficacy testing for cancer immunotherapeutic products. Adoptive immunotherapeutic products which may mediate their therapeutic effect by targeting the tumor directly, such as T cell or NK cell products, have unique challenges in terms of their clinical study design and safety monitoring. A detailed discussion of the clinical considerations of clinical studies for adoptive immunotherapeutic products is beyond the scope of this review. Developers of cancer vaccines planning to initiate studies with adoptive immunotherapeutic products for cancer should discuss the details of the proposed studies with the appropriate review division(s).

### Considerations for early clinical trials

The primary goals of early clinical trials are to assess the safety of the product; to determine the recommended tolerable (Phase 2) dose or optimal biological dose and dosing schedule for the product; and to identify and study the potential biological activities to provide scientific data to guide further product development. The selection of the starting dose and the subsequent dose-escalation scheme, as well as the dosing schedule, for initial clinical trials of a cancer vaccine should be supported by data generated from the preclinical studies and/or prior human experience. When feasible, preclinical *in vitro* and *in vivo* proof-of-concept (POC) studies are recommended to provide the rationale for the proposed clinical trial. The sponsor should provide comprehensive information in the IND, including any existing clinical data regarding the activity and safety profile, to support the safety of the immunotherapeutic product in the proposed trial. The traditional standard dose-escalation schedule in the development of cancer therapeutics uses the so-called “3 + 3 design;” however, a maximum tolerated dose (MTD) is infrequently identified for a cancer vaccine. The dose-toxicity curve may be so flat that the highest dose that can be administered is limited by manufacturing or anatomic issues rather than toxicity. In addition, there may be limited information from preclinical studies to support a starting dose for a monoclonal antibody, e.g., when there is no relevant animal model for the targeted antigen; in such situations, a standard 3 + 3 design may not be appropriate for first-in-human trials. In these cases, the sponsor may consider designing the trial to quickly identify a dose of the monoclonal antibody that is biologically active, while enrolling the least number of subjects to dose cohort levels that are inert. Therefore, this 3 + 3 design may not be the most suitable approach to gathering information from early-phase trials of immunotherapeutic products, and alternative trial designs should be considered. Given the relatively acceptable safety profile of certain types of cancer vaccines (e.g., peptide vaccines) and the desire to expose as few patients as possible to ineffective doses of immunotherapeutic products, alternative dose-escalation approaches, such as accelerated titration (e.g., half-log or two-fold dose-escalation) or continuous reassessment, may be considered instead of the standard 3 + 3 design. When using such designs, the protocol should describe acceptable parameters for the dosing endpoint (supported by data). Irrespective of which dose-escalation approach is chosen, the study protocol should clearly define dose-limiting toxicity (DLT), the subject “off-treatment” criteria, and the study stopping rules that will ensure subject safety. When no DLT is expected or achieved, optimization of other outcomes, such as the immune response, can be useful to identify doses for subsequent studies.

### General considerations for clinical trials

#### Patient population

The conventional model for clinical development of a chemotherapeutic agent involves initial testing in patients with advanced/metastatic diseases and different tumor types to determine the optimal dose, schedule, and MTD. Once its efficacy and safety are demonstrated in the setting of metastatic disease, the same agent may then be developed and tested in subjects who have minimal or no evidence of residual disease [40,41]. However, the time interval from administration of study agent to subsequent disease progression in patients with metastatic cancer may be short. This time may be insufficient for development of an anti-tumor immune response needed for activity/effectiveness of a cancer vaccine. In addition, patients with metastatic disease usually have received multiple treatments (e.g., cytotoxic and/or immunosuppressive chemo- and radio-therapies) for their cancer. These therapies may be detrimental to the immune system, minimizing the potential responsiveness to the cancer vaccine being tested. In contrast, testing cancer vaccines in patients with minimal burden of disease may provide adequate time for the cancer vaccine to elicit a detectable immune response. However, demonstration of efficacy would require following subjects for evidence of disease recurrence, which generally requires a randomized setting. Consequently, developers of cancer vaccines need to weigh the advantages and disadvantages of testing these agents in patients with metastatic diseases versus patients with no evidence of residual disease or minimal burden of disease.

Although it may be acceptable to test heterogeneous patient populations with a common antigen in early-phase trials, this approach may not provide interpretable evidence of efficacy for the purpose of licensure. Interpretation of trial results from a heterogeneous patient population can be challenging, and the objectives of the trials may not be achieved. Thus, in selecting the patient population for cancer vaccine testing in early trials, careful consideration should be given to the heterogeneity of the study population.

#### Monitoring the immune response and immunogenicity

The role of immunogenicity testing in clinical trials is different during the development of cancer vaccines than during the development of immunotherapeutic proteins such as monoclonal antibodies. In cancer vaccine development, immune monitoring is mainly exploratory, especially in early-phase trials, with the major goals to establish proof-of-principle for the proposed pharmacological effect and to show immunogenicity of the administered antigens. However, monitoring of the immune response can be useful for clinical development of therapeutic cancer vaccines to optimize the dose and schedule, determine whether the vaccine induces the intended immune responses, assess immune tolerance, provide proof-of-concept, and aid the decision-making process concerning further product development and later clinical trial design. Mounting a clinically effective anti-tumor response involves a multi-component process coordinated to mediate the effect. Therefore, multiple monitoring assays may be needed to identify and measure the components of the immune responses. Assay standardization should include specific parameters to control for general variability in an immune response across study sites. The assay parameters, such as assay conditions, sensitivity and specificity of the assay, any *in vitro* amplification step involved, positive and negative controls, cutoff values for determining the positive and negative test results from patients’ specimens, and the statistical analytical methods to be used for the test results, should be clearly described in the clinical protocol prior to the initiation of the clinical trial.

In contrast, immunogenicity testing plays a central role in therapeutic protein development. Beginning with the first-in-human trial, development programs of immunotherapeutic proteins incorporate serial testing—patient samples obtained pre-exposure, during administration, and following discontinuation at a time that minimal interference would be expected from remaining immunotherapeutic product levels—to identify anti-product antibodies (APA) and assess any impact that these APA may have on the safety (e.g., hypersensitivity reactions for monoclonal antibody products or autoimmunity for endogenous cytokines), and efficacy of therapeutic proteins [42]. The appropriate role and timing for immune response monitoring and immunogenicity is individualized based on the product characteristics and can be discussed with FDA.

#### Co-development of assays and therapeutics

When the proposed mechanism of action involves a specific antigen or other therapeutic target, consideration should be given to developing an assay or mechanism to measure the target antigen expression in tumor tissues of individual patients and using that information in patient selection or response monitoring. These assays are generally regulated by the Center for Devices and Radiological Health (CDRH). Therefore, sponsors developing cancer vaccines who are considering including the use of an assay with a specific cancer vaccine, should request a meeting with both the relevant FDA product review office and device review division. Discussions begun early in the development process, ideally before submission of an IND and/or IDE (Investigational Device Exemption), may help ensure that product development provides data that establish the safety and effectiveness of the therapeutic product and assay pair.

#### Adjuvants used to stimulate immune response

Cancer vaccine formulations may contain adjuvants, agents that are not generally licensed by themselves but are used in conjunction with vaccine antigens to augment or direct the specific immune response to an antigen. General requirements for inclusion of such adjuvants in licensed biological products are described in 21 CFR 610.15. These requirements include submission of evidence that the proposed adjuvant does not adversely affect the safety or potency of a given vaccine formulation. Information supporting the value of adding the adjuvant should be provided, preferably at an early stage of vaccine development, and may include evidence of enhanced immune response or antigen-sparing effects, and data supporting selection of the dose of the adjuvant. In general, the use of adjuvants is not subject to the requirement that the contribution of each component be demonstrated (as in the case of “fixed combination products” subject to 21 CFR 300.50). However, when products which may have independent clinical activity (e.g., cytokines) are used as adjuvants to enhance the effects of vaccine antigens, the study design and control group(s) should be discussed with FDA. Study design requirements will be considered on a case-by-case basis.

#### Clinical endpoints for cancer immunotherapy studies

One of the most important aspects in designing a late-phase trial is to choose a clinically meaningful endpoint. Demonstrable clinical benefits vary with cancer type and status of disease. Clinical benefits that have supported drug approval have included important clinical outcomes (e.g., increased survival, symptomatic improvement) but also have included effects on established surrogate endpoints. FDA’s “Guidance for Industry: Clinical Trial Endpoints for the Approval of Cancer Drugs and Biologics” dated May 2007, and “Guidance for Industry: Providing Clinical Evidence of Effectiveness for Human Drug and Biological Products,” dated May 1998 contain useful recommendations to be consulted prior to initiating discussions with CBER regarding the endpoints for a late-phase clinical trial of a cancer vaccine. Improvement in how a patient feels, as measured by patient-reported outcome instruments (PRO’s), if properly validated, could constitute a clinical benefit supporting licensure; see “Guidance for Industry: Patient-Reported Outcome Measures: Use in Medical Product Development to Support Labeling Claims,” dated December 2009. While these guidances may be helpful, it is important to keep in mind that endpoints based on radiologic tumor assessments, as discussed in section III.B of the May 2007 guidance, may not be the most appropriate endpoints for a late-phase clinical trial for a cancer vaccine. Recent evidence suggests that cancer vaccines may be more likely to have an effect on overall survival than on other endpoints such as delay in progression or radiological tumor response [43].

#### Disease progression/recurrence immediately or shortly after the initial administration of immunotherapeutic products

In oncology practice, patients are normally taken off current treatment when they have disease progression/recurrence. Because immunotherapeutic products may need time to elicit or amplify an immune response that could manifest as biological activity (i.e., a tumor-specific immune response), a delayed effect can be expected in the subjects who received the vaccine. Shortly after the initial vaccine administration, subjects may experience disease progression prior to the onset of biological activities or effects from the vaccine (delayed effects). Therefore, clinical progression may not be a contraindication to continued administration of immunotherapeutic products. One potential approach to this situation would be for the study protocol to clearly define situations in which vaccination therapy may be continued, for example, when there is no deterioration of subject performance status, subjects continue to meet all other study protocol eligibility criteria, no DLT has been observed, and all toxicities resolved to the baseline level.

#### Concomitant and subsequent therapies

One recent advance in the immunotherapy field is the realization that effective destruction of a tumor involves multiple coordinated immune mechanisms. These mechanisms include, but are not limited to, enhancement of the activities of antigen presenting cells, activation of effector T cells, and removal of suppressor T cells. The ultimate therapeutic effect of immunotherapeutic products may be diminished or enhanced by other cytotoxic or immunomodulatory treatments. Therefore, such cytotoxic or immunomodulatory effects of other treatments should be considered in the clinical trial design and in the overall product development plan. In addition, if the development plan includes concurrent development of two or more novel immunotherapeutic proteins for use in combination, early clinical trial design requires specific considerations because co-development would be expected to provide less information about the safety and efficacy of each individual product. In general, initial clinical trials should be designed to characterize the safety and pharmacokinetics of the individual components—similar to the development program for an individual drug. The proof-of-concept trials should demonstrate the contribution of each immunotherapeutic protein component to the effect of the combination, if not already sufficiently established during the development program [44].

A compelling biological rationale should support use of any concomitant therapy (e.g., chemotherapy, biotherapy, radiotherapy, laser therapy), including the mode of action, dose and schedule of the concomitant therapy, and interactions of the concomitant therapy with the immunotherapeutic product. When standard therapies are available, consideration should be given to the timing and sequencing of these therapies, relative to the schedule of immunotherapeutic product administration, to optimize the evaluation of safety and potential biologic activities. Preclinical exploration of the different options of timing and sequencing of immunotherapeutic product and standard therapy can help guide clinical development. Trial design details, including eligibility criteria and stratification factors, should be carefully considered in order to minimize the impact of standard therapies on the study’s ability to detect the immunotherapeutic product's biological (pharmacodynamic) activity.

#### Statistical issues

The overall clinical effect of an immunotherapeutic product should be evaluated in the context of the currently available therapeutic options. Use of a superiority trial design to demonstrate an immunotherapeutic product’s treatment effect on a chosen endpoint is recommended. In certain clinical settings, the effect size of the available therapy(ies) may be well established. In these limited situations, a noninferiority (NI) trial design and analysis may be considered. However, the design of a NI trial is complex; therefore, early consultation with the FDA and careful consideration of the recommendations in FDA’s “Draft Guidance for Industry: Non-Inferiority Clinical Trials,” dated March 2010 are important.

Adaptive trial designs are considered on a case-by-case basis. Sponsors should consider the recommendations in FDA’s “Draft Guidance for Industry: Adaptive Design Clinical Trials for Drugs and Biologics,” dated February 2010.

Imbalances in subsequent therapies may confound the interpretation of study results, particularly when the primary endpoint is overall survival. Therefore, the study should document the nature and duration of subsequent therapies, and appropriate sensitivity analyses should be pre-specified.

To avoid the biases that can be introduced in the conduct of the trial and in the analyses of the trial results, trials should have appropriate controls, either an active comparator or placebo. Studies involving a placebo should be carefully considered and planned. Withholding an available therapy with proven safety and efficacy may be unethical. However, FDA recognizes that single-arm trial designs may be appropriate in specific situations, such as clinical trials in patients with refractory cancer with no available therapy or rare cancers. The sponsors should consider the limitations of single-arm trial designs.

Blinding of subjects, investigators, and evaluators may be helpful to decrease the risk of bias in the study results. However, either cancer vaccines or co-administered immune stimulatory agents can cause reactions that make the subjects treated with the vaccine easily identifiable. To maintain blinding of treatment assignment, the study may need to provide separate personnel for each of the following: study agent administration; post-administration subject care; and endpoint assessment. For other immunotherapeutic products such as monoclonal antibodies, blinding may be limited due to significant toxicities associated with the administration of these products.

## Conclusions

Immunotherapeutic products, including therapeutic cancer vaccines, monoclonal antibodies, and therapeutic proteins have become part of the standard treatment options of the practicing oncologist, with numerous indications and products licensed by FDA and international regulatory agencies. Many more products are at various stages of development. To facilitate this development, FDA has organized workshops on cancer vaccines and cell and gene therapies, published guidance documents, and organized advisory committee meetings to discuss scientific issues relevant to immunotherapeutic products. Most recently, FDA published a guidance document for cancer vaccine products [37]. It is expected that more guidance documents will be published in the future. With the rapid advances of technology and medical sciences, new concepts and products related to cancer immunotherapy will evolve. The practice of product characterization, preclinical evaluation, and clinical trial methodology will need to adapt to the advances in this exciting field in order to facilitate product development and better protect the public health. Indeed, many technologies and new tools like ‘omics technology, data mining, new analytical assays, stem cell technology, and others are being developed for the evaluation of product characterization and safety. FDA remains actively engaged with the stakeholders in facilitating development of cancer immunotherapeutics and other products for cancer.

## Endnote

^1^Gene therapy products are defined in the 2006 FDA Guidance entitled *Gene Therapy Clinical Trials – Observing Subjects for Delayed Adverse Events*: “Products that mediate their effects by transcription and/or translation of transferred genetic material and/or by integrating into the host genome and that are administered as nucleic acids, viruses, or genetically engineered microorganisms. The products may be used to modify cells *in vivo* or transferred to cells *ex vivo* prior to administration to the recipient”.

## Competing interest

The authors declare that they have no competing interests.

## Authors’ contribution

This review article is a result of contributions made by all authors. RSV, BN and SRH contributed to the CMC section. WH and JL contributed to the preclinical section. PFB, KL, MT and ARD contributed to the clinical and statistical sections. RSV and SRH compiled all sections and prepared drafts of the manuscript. RKP conceived the idea of collaborative article, supervised the development and critically reviewed the article. All authors read and approved the final manuscript.
